# Development of a tool to support general practitioners to help adolescents with knee pain: an analysis using the Theoretical Domains Framework

**DOI:** 10.1017/S1463423623000130

**Published:** 2023-04-03

**Authors:** Clara Guldhammer, Sinead Holden, Alessandro Audreucci, Simon Kristoffer Johansen, Janus Nikolaj Laust Thomsen, Michael Skovdal Rathleff

**Affiliations:** 1 Center for General Practice at Aalborg University, Aalborg, Denmark; 2 Department of Health Science and Technology, Faculty of Medicine, Aalborg University, Aalborg, Denmark; 3 Department of Clinical Medicine, Aalborg University, Aalborg, Denmark

**Keywords:** adolescent, development, focus group, implementation, knee pain, qualitative research

## Abstract

**Aim:**

Using the Theoretical Domains Framework (TDF) and COM-B model, this study aimed to determine the facilitators to a support tool for adolescent non-traumatic knee pain in general practice.

**Background:**

Many children and adolescents with non-traumatic knee pain consult their general practice. Currently, there are no tools to support general practitioners in the diagnosis and management of this group. There is a need to identify behavioural targets that would facilitate further development and implementation of such a tool.

**Methods:**

This study was designed as a qualitative study using focus group interviews with 12 medical doctors working in general practice. The semi-structured focus group interviews conducted online and followed an interview guide based on the TDF and COM-B model. Data were analysed via thematic text analysis.

**Findings:**

One of the biggest challenges from the general practitioner’s perspective was how to manage and guide adolescents with non-traumatic knee pain. The doctors had doubts in their capability to diagnose knee pain and saw opportunity to help structure the consultation. The doctors felt motivated to use a tool but considered access a potential barrier. Increasing opportunity and motivation by creating access in the community among general practitioners was considered important. We identified several barriers and facilitators for a support tool for the management of adolescent non-traumatic knee pain in general practice. To align with user needs, future tools should support diagnostic workup, structure the consultation and be easily available among doctors working in general practice.

## Introduction

One in eight children and adolescents consult their general practitioner annually, due to a musculoskeletal problem (Tan *et al.*, 2018). Non-traumatic knee pain is one of most common reasons for consultation in adolescents with musculoskeletal conditions (Rathleff *et al.*, 2013; Junge *et al.*, 2016). Adolescents with non-traumatic knee pain perceive it as highly important to be given a name/diagnosis and a credible explanation of their pain. A diagnosis is important for the adolescent to cope, accept and adjust to the pain (Yen, 2014; Johansen *et al.*, 2021). Doctors working in general practice are often the first contact. Evidence suggests that being confident and making the right diagnosis is difficult for less experienced/new medical doctors (MDs) working in general practice (Guldhammer *et al.*, 2021). There are evidence-based tools available to support the diagnosis and initial management of adolescent non-traumatic knee pain (Guldhammer *et al.*, 2021), but this research has not yet been implemented in general practice.

Implementation of new treatments are complex and requires in-depth understanding of both individual and collective behaviour in the context where the treatment takes place. Implementation science highlights how changing behaviours or practices in organisations is complex and requires insights into factors influencing behaviours, and knowledge on areas where changes are desirable (Craig *et al.*, 2008; Atkins *et al.*, 2017). Guidance from the UK Medical Research Council on developing and evaluating complex interventions advocates the use of theory to facilitate behaviour change and understand the mechanism of change (Craig *et al.*, 2008; Skivington *et al.*, 2021). In implementation science, theoretical frameworks are one such approach aiming at describing factors believed or found to influence implementation outcomes – for example, how general practitioners can change their behaviour to optimise the management of adolescent non-traumatic knee pain when implementing a new support tool in practice (Nilsen, 2015).

Theoretical frameworks can either be determinant frameworks or evaluation frameworks. The Theoretical Domains Framework (TDF) represents one such framework and was specifically developed to identify determinants of professional behaviour change (Cane *et al.*, 2012; Atkins *et al.*, 2017). The TDF has previously been used in interview studies as a basis for identifying determinants of guideline adherence (Patey *et al.*, 2012; Murphy *et al.*, 2014). Further, the TDF has been implemented in healthcare settings (including primary care) to facilitate understanding of a range of different interventions across the healthcare system (Atkins *et al.*, 2017). The TDF consists of 12 key domains/determinants conducted from 35 different theoretical models of behaviour and includes knowledge, skills, social/professional role, beliefs about consequences, beliefs about capabilities, social influences, motivation/goals, memory attention and decision processes, environmental context and resources, social influence emotion, behavioural regulation, and nature of behaviour (Atkins *et al.*, 2017). An updated version also includes optimism and reinforcement and separates motivation (intention) and goals (Cane *et al.*, 2012). These different domains can be condensed into three core components: capability, opportunity and motivation – forming the COM-B model. The COM-B model demonstrates that human behaviour (B): physical and psychological capabilities (C), social and environmental opportunities (O) and motivators (M) that are reflective (thinking with the head) or automatic (emotional – ‘thinking’ with the heart) are the core elements when addressing human behaviour change (Michie *et al.*, 2011).

Using the TDF and associated COM-B methodological framework, the aims of the study were to identify potential facilitators and barriers in relation to implementation of a future support tool to optimise the management of adolescent non-traumatic knee pain in general practice.

## Methods

### Study design and setting

The study was designed as a qualitative study with online focus group interviews and reflexive thematic text analysis guided by the COM-B model. As the study aimed to evaluate and develop an existing design, we used Action-Research (Bradbury and Lifvergren, 2016) as a methodological framework for guiding our application of methods and aid our identification of points of learning and development of the design throughout the study. Doctors working in general practice participated in two focus groups which were conducted via Microsoft Teams. Focus groups were selected, due to the methods focusing on extracting the convergent views and perspectives of participants taking part, which makes it possible to gather multiple insights into the topic. The inclusion of the COM-B model as an interpretive framework throughout the study allowed us to identify what needs to change in order to effectively change general practitioner’s behaviours. This will help inform how a support tool should function and work in a Danish general practice setting as part of the primary healthcare system in Denmark.

### Ensuring methodological quality through the study

To ensure credibility and transferability, there was a general practitioner (JLT) as part of the research group which supported the lead author (CGV, MD but no GP). This was intended to ensure that the interview guide was of relevance to GPs in general. To heighten credibility and confirmability, the senior author (MSR) made a check of 20% of all coding and supported the analysis to confirm it genuinely reflected the transcripts of the interviews. General practitioner, JLT, performed an external check of subsequent analyses and conclusions (as did the other researchers in the author group) and to ensure they were not strongly biased.

### Inclusion and exclusion criteria for participants

We included 12 doctors in two separate focus groups. We considered this as an adequate number of doctors and focus groups to assess our outcomes in this study (Hennink *et al.*, 2019). We recruited doctors through the regional association responsible for continued professional development in general practice called Nord-KAP (Kvalitetsenheden for Almen Praksis i Region Nordjylland), the Center for General Practice at Aalborg University reference group, and through the research team’s professional network.

To be eligible, potential participants needed to be doctors working in a general practice setting either as a specialist in general medicine, as part of the postgraduate specialist training programme, or as part of the clinical basis education programme (KBU) for younger doctors in Denmark. We included doctors both with and without an interest in sports medicine. Further, we aimed to include both doctors with many years of experience and those with less experience in general practice.

All participants gave their informed consent to participate before the focus group interviews. Once included, participants completed a questionnaire regarding sex, age, geographical location, experience as a doctor (in years), and whether they had special interest in sports medicine or research. Ethical approval was waived by the committee of North Denmark Region due to the studies low-risk nature.

### Interview guide and Theoretical Domains Framework

**Figure 1.**
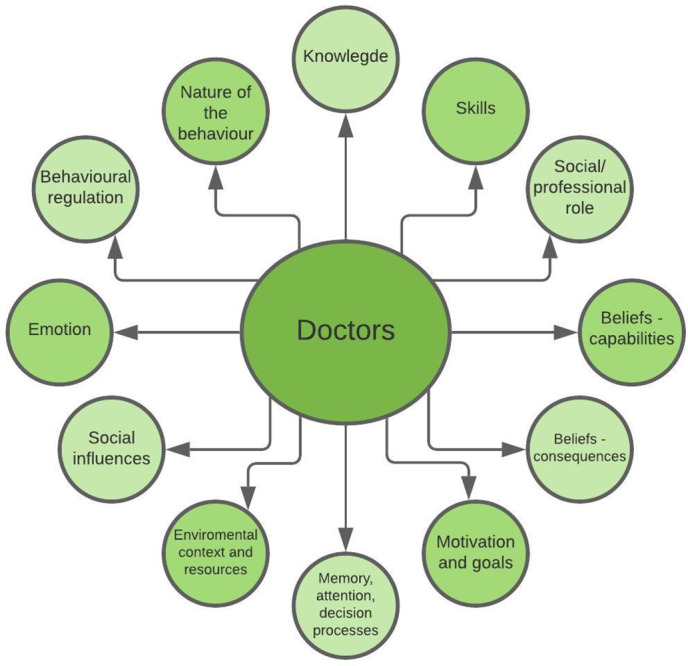
Key domains covered in the topic guide formed by the TDF and associated COM-B framework

In this study, we use the TDF to form the interview guide. When the outcome in this study is to analyse facilitators and barriers to implement our support tool, determinant frameworks, as the TDF, are adequate (Nilsen, 2015). Determinant frameworks aim to describing general types of determinants that are hypothesised or have been found to influence implementation outcomes, where each type of determinant typically comprises a number of individual barriers or facilitators that impact the given outcome (Nilsen, 2015; Atkins *et al.*, 2017). Based on this, an interview guide was designed with open and probing questions, intended to cover the domains of the TDF (Atkins *et al.*, 2017) (see Additional File 1). Each domain was broached with an open question to elicit the first response and provide space for participants to articulate their perspectives, followed by a series of follow-up questions to gain deeper insights based on the informant’s responses. Figure 1 shows the key domains covered in the topic guide. For the focus groups, doctors working in the same general practice were separated into different groups.

### Procedure

We included 12 doctors which were divided into two focus group (six doctors per interview) based on an aim to create maximal variation of years of experience and special interest in adolescent knee pain. The focus groups were moderate by the lead author (CV) and were conducted using Microsoft Teams and recorded using a recorder. Care was taken to ensure that both focus groups were conducted the same way to improve the comparability between the two groups. The focus group interview was initiated by CV by giving a short introduction about adolescent knee pain and the aim of the focus group. The ‘raise hand’ function of Microsoft Teams was not used as it was not deemed relevant due to the relative ‘small number’ (six doctors per focus group interview) of people participating. Participants raised their physical hand, and CV moderated the discussion using the interview guide.

### Data analysis

Characteristics for recruited doctors were calculated through descriptive statistics using Stata/MP 16.0 for Mac.

The data collected during focus group interviews were analysed using the Reflexive Thematic Analysis approach by Braun and Clarke (Braun and Clarke, 2019), which included transcriptions, familiarisation, coding, identification of themes, reviewing themes, refining themes and reporting themes in a narrative. We included domains from the TDF and associated COM-B framework to inform our identification, organisation and refining of themes via deduction.

The interview recordings were transcribed by CV in Microsoft Word in accordance with Braun and Clarke (2019) guidelines for ensuring meaning retention. The transcripts were validated against the audio recordings to check for accuracy of the transcriptions.

The lead author (CV) first read through the entire set of transcripts through NVivo to familiarise herself with the contents of the transcribed texts. Subsequently, lead author CV identified and coded all statements to generate preliminary codes informed by the COM-B and TDF framework. To test the quality of the coding, 20% were sampled and checked by the second author (MSR) (Thomas, 2006). Any discrepancies were discussed and recoded until consensus. The codes were then checked again to ensure consistency. A secondary round of open-scope coding was conducted to identify codes which were not included in the COM-B model. After initial coding and checking, codes were categorised and merged into themes informed by the TDF model to allow for external themes to form as well (Supplemental File 2). This required CV to re-read data within the codes before allocating the codes to the specific domains, thus progressively refining the themes with each reading. Finally, the themes were defined by eliminating overlaps and redundancies and named by mapping them to domains of the COM-B model where applicable. See Supplemental File 2 for the description of coding.

## Results

### Characteristics of doctors

The focus group interviews included MDs with a range of experience with non-traumatic adolescent knee pain (Table 1). Only one of the doctors had a special interest in sports medicine and specialised knowledge on management of adolescent non-traumatic knee pain in general practice. Table 1 describes baseline characteristics of doctors participating in the focus group interviews.

**Table 1.** Baseline characteristics of doctors participating in focus group interviews

Baseline characteristics	Doctors participating in focus group interviews
Number of participants (*n*)	12
Age (mean ± SD)	40.4 ± 3.7
Sex (female, %)	58.3 (n = 6)
Clinical experience in general practice (years)	
0–5 years (%)	58.3 (n = 7)
5–10 years (%)	25.0 (n = 3)
10–15 years (%)	0 (n = 0)
15–20 years (%)	0 (n = 0)
+20 years (%)	16.7 (n = 2)
Special interest in research (yes, %)	50 (n = 6)
Special interest in musculoskeletal pain including sports medicine (yes, %)	25 (n = 3)

#### Coding of data

Using a two-step analysis approach, 42 unique codes were identified in the data set. First, an initial deductive coding was done using the TDF and COM-B elements to identify preliminary themes related to the model. After the deductive coding, the second open-scoped inductive coding was conducted to identify themes outside the concepts, progressively refinement and naming of themes (deductive) in relation to the COM-B model and to identify the final themes presented in the results. All codes could be applied to at least one domain of the TDF and COM-B model. Several codes were mapped to two or more domains from the TDF, which generated 12 main themes (see Additional File 3 for an overview of the coding process).

### Data analysis using the TDF and COM-B model

Overall, doctors were receptive to a support tool to optimise the management of adolescent non-traumatic knee pain in general practice. The doctors believed that such a tool would be relevant and interesting to use in the future. How the results were mapped to the COM-B model are described below. See Table 2 for summary of the results.

**Table 2.** Summary of the results

COM-B	TDF	Summary of the results from the focus group interviews
Capability	Skills Knowledge and nature of behaviour Memory, attention and decision processes Behavioural regulation	The doctors were familiar with a few different diagnoses. Doctors’ self-assessment of their capabilities varied. One of the clearest challenges for the doctors was how to manage, help and guide the adolescents. A challenge for the doctors were especially the physical examination, which could be a target for the support tool. The doctors believed that the support tool could improve their self-confidence. The doctors believed they could improve their ability to differentiate between the benign diagnoses and more serious diagnoses. The doctors had clear opinions about the design of the tool – it should be inviting, interesting and short.
Opportunity	Environmental context and resources Social influences	The doctors did not have any concerns about possible negative effects of using the tool. All doctors believed that the tool would not affect the control in the consultation in a negative way. Knowledge of new developments is often reached through colleagues, but personal opinions to the use of the tool would be more important than collegial approval.
Motivation	Professional role and identity Beliefs about capabilities Beliefs about consequences Goals, intention, motivation and emotions	An important facilitator to successful implementation was promoting through ‘channels’ used among general practitioners. The most important barrier to successful implementation was availability. Doctors saw themselves as important in the management of adolescent non-traumatic knee pain – especially to diagnose and exclude serious cases and become a safety net for the patients. Emotionally, the doctors found the tool interesting and relevant. The doctors would be motivated to use the tool if it showed impact on their clinical practice.

### Capability

#### Skills, knowledge and nature of behaviour

Doctors were familiar with some diagnoses of adolescent non-traumatic knee pain. They all showed a good understanding of the consultation process. The doctors agreed that questions focusing on training and activity load are important to clarify in the medical history. However, there were specific areas where doctor’s skills and knowledge could be targeted to improve management. For example, a common challenge for the doctors was the physical examination – especially when the physical examination did not end up with conclusive findings compared with the patient history. One doctor described his challenge like this:

*‘ (.) and when they consult me, I experience that I cannot find anything in the physical examination. They tell me, that they have pain inside the knee, but when I examine them, I cannot really find anything. In such cases I am often very challenged.’* (Doctor 7).

Most of the doctors felt incapable in the physical examination, in relation to non-traumatic knee injuries. Some doctors stated that they use the same physical examination for both non-traumatic and traumatic knee injuries, which reflects a knowledge gap. The knowledge gap was evident in the statement below:

*‘Actually, when I think about it, I use the same knee examination for all kinds of knee pain – so I think I find it difficult to find out how I examine the non-traumatic knee injuries (…), because it is perhaps obvious that if I examine the knee in relation to find cruciate ligament or meniscus injuries then it is just like every other knee examination and not specific for non-traumatic knee pain. (…) It is obvious that I probably don’t find anything on my knee examination with the same strategy I do as standard.’* (Doctor 5).

The more experienced doctors were more confident in their capabilities to manage adolescents with non-traumatic knee pain. However, some doctors expressed doubts surrounding their capabilities for management in practice and felt they often refer to physiotherapy instead. The younger doctors were more likely to refer to physiotherapy or secondary care compared to the more experienced doctors as highlighted in this statement.

*‘I think the balance is to take these types of patients serious. In my opinion we have a lot of these patients with knee pain, where we find nothing in the physical examination and then the advice, we tell them, does not feel like strong evidence, but is more like a chit chat’* (Doctor 3).

#### Memory, attention and decision processes

Confidence was identified throughout the analysis of an important theme. The doctors believed the support tool could improve their confidence during consultations with adolescent non-traumatic knee pain. Increasing their knowledge about the management strategies to adolescent non-traumatic knee pain was identified to increase confidence during the consultation and thus could support automatic decision-making as indicated in this statement:

*‘I believe that the more I know about this, the more confident I am, and this is reflected in how confident I seem to be in my contact with the patient’* (Doctor 9).

A very important point from one of the doctors was that a support tool could help the doctors to improve their ability to differentiate between the benign diagnoses and the more serious/malignant diagnoses. This statement was acknowledged as very important and beneficial from the other doctors:

*‘I think that the better you are to diagnose and differentiate the benign diagnoses, the better you are to differentiate and diagnose the malignant and more serious diagnoses and distinguish them from the benign cases. I believe that the tool could improve our ability to diagnose the malignant cases that we are not allowed to miss in the clinic’* (Doctor 3).

#### Behavioural regulation

Younger doctors wanted support and guidance to the preliminary part of the consultation, the patient history and physical examination, where the more experienced doctors highlighted guidance to treatment and plan. The doctors felt that the tool should contain guidance to the entire consultation process, so it is accessible to everyone regardless of personal strengths and limitations. Furthermore, doctors wanted guidance on what could be the focus of the first consultation and what could be the focus of the second consultation, if any, to remind the doctors of the option of the second consultation. Also, the doctors requested information on evidence-based advice to be given to the patients. This way they would feel empowered regarding guidance for their patients and less reliant on referral to physiotherapist or secondary care as indicated below:

*‘Overall three things: Key questions I have to remember in my medical history – for example questions related to the pain localization, red flags and how I can differentiate between the different diagnoses. What I should focus on in the physical examination and last, something that unites the information from the medical history and physical examination and then a treatment or guide I can tell the patient – I think this could be a great tool’* (Doctor 5).

The doctors had clear opinions about how such a tool might look and be designed. It should be inviting, interesting and short, precise, and easy to use and memorise. They emphasised that design and layout were very important factors. The tool should both be in a paper version and an online version.

### Opportunity

#### Environmental context and resources

The doctors did not have any concerns about possible negative effects of using the tool in a general practice setting. All doctors believed that the tool would not affect the control in the consultation in a negative way. Instead, they believed that the tool could have a positive impact on the consultation and the consultation process. As highlighted above, the doctors believed that by helping structure the entire consultation, this would give them the opportunity to change their normal practice when seeing patients with knee pain. Many of the doctors were familiar with using tools to support them in their daily practice. The doctors had no concerns about the safety of using the tool. As highlighted in this statement, they shared the opinion that ultimate clinical responsibility would still be theirs, regardless of presence of whether they were aided by a support tool.

*‘The tools we are used to are intended to support us and give us an overview. That does not exempt us from not using our clinical judgement, as doctors, or be in doubt and refer to further examination. We all know that, even though a new support tool is implemented in our practice, it is still our responsibility to look out for the ‘zebra’ – So, I don’t think that the tool will limit our clinical practice’* (Doctor 3).

#### Social influences

Doctors said that knowledge of new developments is often reached through colleagues; however, when it came to the question of whether collegial approval would affect their decision to use the tool, they indicated that personal opinions would be more important to increase automatic motivation.

### Motivation

#### Doctors – professional role and identity

Doctors believed that their role in the management of adolescent non-traumatic knee pain is related to their usual role as general practitioners – being able to distinguish between who is sick and who is healthy. Doctors thought they were important to diagnose and exclude serious cases and become a safety net. One doctor believed there was a very low prevalence of these patients in general practice. As indicated in the statement below, this might indicate that there is a need to target doctors’ concepts surrounding the prevalence of knee pain in general practice and the important role GPs play in treating these patients.

*‘I may think we have a minor role in this management of adolescent non-traumatic knee pain compared to what we could – due to the fact that a lot of adolescents seek help elsewhere, deal with it themselves, or don’t deal with it at all. Maybe, and I just reflect, your support tool is not implemented in the right setting as general practitioners only help approximately 10% of adolescents with non-traumatic knee pain.’* (Doctor 7).

#### Beliefs about capabilities and facilitators to implementation

Facilitators included communicating and delivering the tool through ‘channels’ commonly used among general practitioners, that is, local and national professional journals and popular press, association with local and national associations for general practitioners, availability of training courses in use of the tool during continuing education, etc. The doctors highlighted that availability was very important – the tool should be accessible.

#### Beliefs about consequences and barriers to implementation

The doctors were worried about forgetting the tool due to the low prevalence of adolescent non-traumatic knee pain in general practice. The doctors disagreed about ‘time’ as a barrier. Some of the doctors thought that the time limitation in general practice was very important to take into consideration, while others thought that consultations would eventually become more time efficient using the tool. An interesting barrier raised during the interviews was that some more experienced general practitioners with ingrained opinions could be less receptive to change and implementing new ways of practice compared to younger doctors:

*‘I think there might be some ingrained opinions among general practitioners. You must be certain to reach all types of doctors because I am pretty certain that we younger doctors new in general practice seek a lot of knowledge and would be quick to find such a tool if it appears in searches, but with more experienced doctors with a lot of ingrained knowledge it would perhaps be harder to reach them and tell them that things can be done differently’* (Doctor 5).

#### Goals, intentions, motivation and emotions

Emotionally, the doctors found the tool interesting and relevant. Their interest in such a tool grew over the course of the focus group interview, as participants reflected upon and became aware of their own diagnostic practices. The doctors would be motivated to use the tool if results from high-quality studies in the implementation process showed good results with clinical impact on the management of adolescent non-traumatic knee pain.

*‘I haven’t required a tool like this before you contacted us to be a part of this group discussion, but after our discussion today, I reflect that a lot of us think our management is quite unstructured and in relation to this I find your tool very relevant to help with the structure and guide us how to manage adolescents with non-traumatic knee pain. I think it will have an impact on the patient and us as well’* (Doctor 3)’.

## Discussion

This study used the TDF and associated COM-B model to identify barriers and facilitators for implementing a tool to support general practitioners in the management of non-traumatic knee pain in adolescents. The analysis showed that GPs saw benefit in such a tool, but several barriers and facilitators were uncovered that need to be addressed during development of a potential future tool. A specific facilitator would be to support the GPs in the physical examination, which was often a challenge for them, as well as increasing their knowledge on potential treatments/evidence-based advice to prevent the need to refer them on. Furthermore, a potential barrier emerging from the interviews was that the tool needed to be disseminated through well-known (and respected) ‘channels’ in the general practice environment in order to facilitate the use of the tool.

### Findings in context

An important target for implementing such a support tool would be to guide and help the doctors structure the consultation and the physical examination. It is well documented that diagnosing and managing adolescents with non-traumatic knee pain can be challenging (Nunes *et al.*, 2013; Michaleff *et al.*, 2017). Previous research suggests that only one in five diagnoses of non-traumatic knee pain given by junior doctors is correct (Guldhammer *et al.*, 2021). Recent evidence highlights that a specific diagnosis and a thorough explanation is one of the most important aspects of the initial consultation. This highlights the relevance of supporting the diagnostic workup. Furthermore, a lack of specific diagnosis may result in a heterogeneous treatment strategy. The doctors highlighted that they felt incapable in their practice in relation to non-traumatic knee pain, and that they often focus on examining the knee for traumatic injuries. This is consistent with previous research showing that non-traumatic knee injuries often are overlooked compared to traumatic knee complaints (Rathleff *et al.*, 2013; Junge *et al.*, 2016). This highlights the need for increased knowledge and skills in relation to managing adolescent non-traumatic knee pain.

Ingemansson et al investigated practice guidelines in the context of primary care (Ingemansson *et al.*, 2014). They found that general practitioners perceived practice guidelines as a support to deliver high-quality care while also giving them confidence in the treatments they delivered. In our analysis, they stated that a facilitator for implementation was the prospect of the tools ability to empower them and help them deliver high-quality care. The doctors believed that the support tool could improve their self-confidence and their ability to diagnose and differentiate between the different diagnoses, which makes it important that the support tool target their knowledge gap (diagnostic clarification) and their skill gap on the physical examination. But the doctors emphasised the importance of having confidence in using the support tool to change their behaviour. If the doctors did not build confidence in using the support tool, they saw it is a barrier to use the support tool. By targeting these knowledge and skill gaps in our tool, we can directly target the doctors’ needs which increases the probability of changing behaviours according to the COM-B model of behavioural regulation. As outlined in Table 3, there are more than one intervention which can target these needs, and the design and delivery of the tool present a feasible approach to ensure that important aspects are targeted. An important facilitator (or potential barrier) was the design and layout of a tool. The doctors had strong opinions about the need for the tool to be inviting, interesting and short, precise, and easy to use and memorise. This is similar to the results from Ingemansson which showed that general practitioners have a desire for support tools to be short, concise, with a clear overview and a pedagogic layout (Ingemansson *et al.*, 2014).

**Table 3:** Potential interventions and examples of how these can be applied based on the behavioural targets identified in the focus groups

Intervention function	Definition	Example
Education	Increase knowledge or understanding	Provide information on diagnoses and best evidence for advice for management
Environmental restructuring	Changing physical or social context	Make tool accessible in general practices and through ‘channels’. Make reminders on notice boards, so they remember to use the tool,etc.
Training	Imparting skills	Skills on clinical examination
Environmental restructuring	Changing the physical or social context	- Prompts to ask for second consultations - Cues for structuring the consultation Accessibility of the tool
Enablement	Increasing means/reducing barriers to increase capability or opportunity	Increasing time for consultations Accessibility of the tool

### Limitations

This study had several limitations which readers should consider when reading the results. By drawing upon the domains of the TDF framework and associated COM-B model to inform the design of an interview guide, as well as guiding our thematic analysis, our scope of inquiry was directed towards identifying potential barriers, facilitators and areas of improvement for future iterations of the tool. To safeguard for conceptual circularity, our data were subjected to open-scope coding and review by the second researcher to identify new topics prior to formulating themes (Dana and Dumez, 2015). Still, the narrow scope nature of this study, the inductive approach and reliance of theory meant that some degree of reproduction was deemed acceptable due to the specific and narrow nature of our research question. Due to COVID-19 restrictions in Denmark, the focus group interviews were conducted virtually using Microsoft Teams. As the decision to switch to a digital set-up provided several benefits like, for example, access to participants, reduced transport time for participants and recording options (Shamsuddin *et al.*, 2021), our digital set-up may impact the dynamics, fluency and depth of participants discussions (Kite and Phongsavan, 2017). Yet, as previous studies have documented how digital focus groups can produce high-quality insights despite the slower pace (Abrams *et al.*, 2015), we south to amend the constraints imposed by the media by being mindful of these when facilitating the interviews (Kite and Phongsavan, 2017). Finally, we were aware that focus interviews include risks of say-do problems and social desirability bias due to individuals desire to conform to align with the views of their peers (Bergen and Labonté, 2020) and how asking participants to disclose challenges related to their treatment of patients might enhance this further. Having only GPs present, guaranteed anonymity and creating a pleasant atmosphere were prioritised to ensure the interviews became a safe space for GPs to discuss their challenges related to diagnosing adolescent knee pain.

### Implications for research and/or practice

This study contributes with the first set of considerations on the development and implementation of a support tool for managing adolescent non-traumatic knee pain in general practice. Further, the analysis revealed a need among doctors working in a general practice setting for such a tool and provided initial anchor points for design. Other areas of MSK research have benefitted from support tools to help facilitate the delivery of targeted treatment (Hill *et al.*, 2011). Future studies are needed to understand if such a support tool for adolescent non-traumatic knee pain may be developed into a tool which may also help to deliver targeted treatment.

## Conclusion

We identified factors which need to be addressed in the design and implementation of a support tool for managing adolescent non-traumatic knee pain in general practice. Many interventions are available to target the identified needs (Table 3). To enable behaviour change, the design and delivery of a support tool should target the clinical exam, diagnosis and consultation structure. We have developed a tool for assisting the diagnosis, and incorporating these aspects prior to implementation may increase the uptake and use. Future research is needed to facilitate this together with stakeholders prior to implementing in general practice.

## Supplementary material

For supplementary material accompanying this paper visit http://doi.org/10.1017/S1463423623000130. click here to view supplementary material
