# Supplementary material for: Development of a tool to support general practitioners to help adolescents with knee pain: an analysis using the Theoretical Domains Framework
Source: Prim Health Care Res Dev. 2023 Apr 3;24:e24. doi: 10.1017/S1463423623000130 (PMC10156466; doi:10.1017/S1463423623000130)
Supplement: Supplementary file 1 [file S1463423623000130sup001.docx]

# Appendix

### Additional File 1 – Interview guide

| **Prompts for focus groups according to Michie’s theoretical domains (TDF).** | |
| --- | --- |
| **Theoretical domain** | **Interview questions/prompts** |
| Knowledge | What do you know about adolescent non-traumatic knee injuries? Which different diagnoses are you familiar with and what differentiates them?  How is your current practice in a consultation with an adolescent with knee pain?  What challenges do you have in such a consultation?  Are there parts of the consultation process where you feel uncertain?  Do you have any thoughts about whether there are adolescents who need follow-up consultations? What do you think can affect a good prognosis? |
| Skills | What considerations do you have when an adolescent seeks help due to knee pain with a non-traumatic origin?  How did you learn to manage non-traumatic knee pain in adolescents?  Have you changed the way you manage knee pain in adolescents during the years? And how?  What do you think of your skills in relation to management of adolescent non-traumatic knee pain? Do you think you can improve your management strategy?  From your experience, are there any factors that might influence the management of adolescent knee pain that is frequently not considered?  Do you know about factors that might affect optimal management of adolescents with non-traumatic knee pain? |
| Social/professional role | What kind of role do you see yourself in as a doctor in relation to the management of non-traumatic knee pain in adolescents?   - Why do you think you have that role? - Do you think a support tool could help the clinical consultation?   As a doctor, how do you think you influence adolescents with knee pain? Do you think they always follow your recommendations? |
| Beliefs about capabilities | What needs do you consider in order to increase your ability to diagnose and treat adolescents with non-traumatic knee pain? – what facilitators and barriers do you see when using the tool to meet those needs?  Do you think a support tool will increase your diagnostic accuracy and improve the management of adolescent non-traumatic knee pain?  How do you think such a tool will affect your control/ management in relation to the consultation? |
| Beliefs about consequences | What thoughts do you have in relation to the content of the tool, so you can use it safely in the consultation?  Would you be worried about overlooking relevant and important information in your medical history if you use the tool?  What can help the implementation of the tool to be successful and used by you in the clinic in the future?  What barriers do you see for such a tool to be used/implemented in general practice? |
| Motivation and goals | What will motivate you to use the tool?  Would you be inclined to use a strategy that goes against your clinical experience if advised by the tool?  What do you expect from a support tool if you should use it in your consultation? Do you think you will use it?  What goals must the support tool meet if you should use it in your consultation? |
| Memory, attention and decision processes | How do you think the support tool will affect your decision-making process when having consultations with adolescents with knee pain? |
| Environmental context and resources | What opportunities and challenges do you see with using the tool within the framework of a normal consultation in general practice - e.g., context of the GP consultation? |
| Social influences | Would you be more likely to use the tool if you know your colleagues also use it?  How do you think your colleagues see / think about you if you use the tool? |
| Emotion | What thoughts and feelings/emotions do you associate with the fact that we want to implement a new tool for optimal management of adolescent non-traumatic knee pain in general practice? |
| Behavioural regulation | What does it take for you to start using the tool in your consultations? What should the tool contain? / are there pitfalls where you think that "if x, then I won’t use the tool”  Do you have any preferences regarding layout? Paper version? App? Online? Etc. |
| Nature of the behaviour | What experiences do you have with management of adolescents with non-traumatic knee pain in general practice? - Do you experience a general need for a support tool in that context? |

### Additional File 2 – Description of coding

| **Description of coding** | | |
| --- | --- | --- |
| **COM-B Domain** | **TDF** | **Themes coded under this node** |
| Psychological CAPABILITY | Knowledge and nature of behaviour | The doctors’ understanding and knowledge about adolescent non-traumatic knee pain, including:  Understanding of origin, causes and management of adolescent non-traumatic knee injuries  The different diagnoses and how to differentiate between them  The prognostic factors  Current management of adolescent knee pain  The challenges that doctors might meet in a consultation with adolescent non-traumatic knee pain  Parts of the consultation where the doctors feel uncertain  Experience with adolescent non-traumatic knee pain |
| Psychological CAPABILITY | Memory, attention and decision processes | How the tool affects the doctor’s decision-making-process  The integration of the support tool into the consultation process |
| Psychological CAPABILITY | Behavioural regulation | Preferences to layout, format and the content of the tool to change behaviour  External factors, availability to clinics and doctors  Opinions about how to implement this tool |
|  |  |  |
| Physical CAPABILITY | Skills | Considerations and reflections when doctors see adolescents with non-traumatic knee pain  How the doctors have learned to manage adolescent non-traumatic knee pain  How they have changed/ improved the management during the years  Doctors opinions about their skills to management of adolescent non-traumatic knee pain  Factors that doctors have experienced can affect the management of adolescent non-traumatic knee pain |
|  |  |  |
| Social OPPORTUNITY | Social influences | Stigma and thoughts from colleagues in relation to using the tool  Group behaviour and norms  Positive and negative influences from others |
|  |  |  |
| Environmental OPPORTUNITY | Environmental context and resources | Opportunities and challenges in relation to using the tool in a GP consultation framework |
|  |  |  |
| Motivation REFLECTIVE | Beliefs about capabilities | The tool’s role in diagnosis and treatment  The tool’s influences on self-confidence  The tool’s influence on control in the consultation  Facilitators in the implementation of the tool in general practice |
| Motivation REFLECTIVE | Motivation/intention and goals | Motivation to use the tool  Motivation to change and accommodate new knowledge  Expectations from doctors to the tool  Goals about personal improvement from using the tool |
| Motivation REFLECTIVE | Beliefs about consequences | Beliefs about the trustworthiness of the tool – content of the tool to ensure trustworthiness  Clinical impact of the tool  Fears of missing important information when using the tool  Barriers to the implementation of the tool in clinical practice |
| Motivation REFLECTIVE | Identity – social/professional role | Beliefs and opinions about role of doctors in management of adolescent non-traumatic knee pain  Role of being a general practitioner  Responsibility of being a doctor  Beliefs about doctor-patient relationship |
|  |  |  |
| Motivation AUTOMATIC | Reinforcement/habit | Experience with adolescent non-traumatic knee pain  What the doctors think about the simplicity of the tool – the importance of simplicity of the tool |
| Motivation AUTOMATIC | Emotions | Opinions, thoughts and reflections in relation to this new intervention – what the doctors think of the development of the tool |

## Additional File 3 Mapping of codes to themes from Theoretical Domains Framework (TDF) and COM-B model

** mapped to two different themes from the TDF. *** mapped to three different themes from the TDF.

| **Code assigned directly to transcripts from focus group interviews** | **Themes from TDF** | **COM-B model** |
| --- | --- | --- |
| Knowlegde  Prognostic factors  Social factors**  Prevalence of adolescent knee pain  Physiotherapy  Communication  Current practice  Medical history  Clinical examination  Challenges  Uncertainty (doctors) | Knowlegde | Phycological CAPABILITY |
| Impact of the tool**  Control of the consultation using the tool** | Memory, attention and decision processes | Phycological CAPABILITY |
| Design and layout of the tool**  Content of the tool**  Red flags  Revision and update of the tool | Behavioural regulation | Phycological CAPABILITY |
| Follow up consultations  Social factors**  Parents  Expectations – child vs. parent  Difficult/challenging age group  Patient views  Experience – doctors  Self-assurance – doctors | Skills | Physical CAPABILITY |
| The importance of the popularity of the tool | Social influences | Social OPPORTUNITY |
| The tools control of the consultation**  Impact of the tool***  Concens about overlooking important information**  Availability**  Challenges – using the tool** | Environmental context and resources | Environmental OPPORTUNITY |
| The tools control of the consultation**  Experience with using support tools in practice  Gathering knowledge  Impact of the tool***  Increased self-confidence when using the tool  Communication through “channels”  Education  Facilitators to implementation | Beliefs about capabilities | Motivation REFLECTIVE |
| Motivation  Impact of the tool***  Evidence | Professional role and identity | Motivation REFLECTIVE |
| Availability**  Concens about overlooking important information**  Challenges – using the tool**  Barriers to implementation  Clinical impact of the tool | Motivation and goals | Motivation REFLECTIVE |
| Responsibility – doctors  Opinion about general practice  Role as a general practitioner | Beliefs about consequences | Motivation REFLECTIVE |
| Content of the tool**  Design and layout of the tool** | Reinforcement/habits | Motivation AUTOMATIC |
| Opinions about the tool | Emotions | Motivation AUTOMATIC |
